# Stability of buried and networked salt-bridges (BNSB)in thermophilic proteins

**DOI:** 10.6026/97320630015061

**Published:** 2019-02-03

**Authors:** Amal Kumar Bandyopadhyay, Rifat Nawaz Ul Islam, Debanjan Mitra, Sahini Banerjee, Arunava Goswami

**Affiliations:** 1 Department of Biotechnology, University of Burdwan, Burdwan, West Bengal,India; 2 Department of Zoology, University of Burdwan,Burdwan, West Bengal,India; 3 Department of Biological Sciences, ISI, Kolkata, West Bengal,India

**Keywords:** Thermophilic protein, stability, networked salt-bridge, buried, desolvation cost, novel method

## Abstract

Thermophilic proteins function at high temperature, unlike mesophilic proteins. Thermo-stability of these proteins is due to the unique buried and networked salt-bridge (BNSB). However, it is known that the desolvation cost of BNSB is too high compared to other favorable energy terms. Nonetheless, it is known that stability is provided generally by hydrophobic isosteres without the need for BNSB. We show in this analysis that the BNSB is the optimal evolutionary design of salt-bridge to offset desolvation cost efficiently. Hence, thermophilic proteins with a high level of BNSB provide thermo-stability.

## Background

Thermophiles thrive at high-temperature (~100 °C), which is similar to other extremophiles that live under extreme of physical and chemical conditions. Compare to mesophiles, extreme proteins incorporate additional strategies for the maintenance of the structure, stability, and functionality [1,2,3,4,5]. It is not like a tolerance, but an evolutionarily transformed situation, where these environments have been obligatory for their optimal growth and functionality. Inside the cell, the conditions are also similar to the outer environments. Using orthologous enzymes from thermophiles and mesophiles, it has been demonstrated that the optimal activity is only obtained when the temperature of the reaction mixture is made similar to their respective ecosystems [6]. Homologous positions of functionally identical proteins from thermophilic archaea and mesophilic bacteria showed ~75% difference. Thermophiles maintain structure and stability of their proteins in their ecosystem, when it is known that under extreme conditions, dielectric constant of the solvent in the cytoplasm [7,8], subunit association and dissociation equilibria, surface tension, solubility and stability of mesophilic proteins are drastically affected [9,10,11,12].

The net and component energy terms of salt-bridge are computed by isolated-pair method (IPM) [13,14]. Net interaction energy (ΔΔGnet) of a given salt bridge is composed of the bridge (ΔΔGbrd), desolvation (ΔΔGdslv) and background (ΔΔGprot) energy terms. ΔΔGbrd, which is the direct and pH-dependent term, is always contributing. ΔΔGdslv, on the other hand, is related to the desolvation of charge-partners and thus, it is always costly. The ΔΔGprot, which depends on the interaction of partners of salt-bridge with other charges of the protein, could either be contributing or costly. The latter two are indirect and pH-independent terms. Thus, stabilizing and destabilizing effects largely depends on location, microenvironment, and geometry of salt bridges [13,14]. Double mutation cycle and pKa approaches are popularly used for experimental measurement of the energy of salt-bridges. Unfortunately, none of these methods could determine the indirect terms and hence the ΔΔGnet and thus, the computational method is the only choice for the purpose [15].

Because desolvation cost of buried salt-bridge is much higher than that in the surface [16], and because such an effect has been highlighted in one computational study, it has been anticipated that the chance of getting stable buried salt-bridge is less and thus, protein that harbors such salt-bridges, could be redesigned better by replacing these salt-bridge residues by the use of hydrophobic isosteres [17]. Formation of buried salt-bridge is at the rate-limiting step of protein folding [18], as large desolvation cost is involved in hiding charged partners of salt-bridge in the protein interior [19]. However, it has to be borne in mind that hydrophobic force is severely affected under extreme solvent conditions as dielectric constant is reduced to ~45-55 [7,11]. In turn, in a low dielectric medium, the strength of electrostatic interaction increases [16]. Yet, highly stable, buried and networked salt-bridges are much higher [11,13] than hydrophobic residues in extreme proteins.

In this work, we use Poisson-Boltzmann Equation (PBE) and it solver method i.e. APBS [20] along with PDB2PQR [21] to estimate ΔΔGnet and associated energy terms (ΔΔGdslv, ΔΔGprot, and ΔΔGbrd) of some networked and deeply buried salt bridges of thermophilic protein to check their level of stability. In this computation, instead of using conventional IPM [13,14], we employ a new protocol (Network unit method i.e. NUM) for obtaining energy terms of networked salt-bridges. This methodological improvement allows us to show as to how sufficiently the high cost of ΔΔGdslv is offset by these salt-bridges. We then discuss our results in the light of others to highlight the advantage of formation of networked saltbridge in the core in general and in thermophilic proteins in particular. Taken together, we believe our method may provide a scientific explanation as to how desolvation cost is bypassed by buried and networked salt-bridges.

## Methodology

### Dataset

In this work, we used 5T88 for obtaining networked and buried salt bridges. The crystal structure was obtained from Research Collaboratory for Structural Bioinformatics (RCSB) protein data bank (PDB) [22]. The structure was then minimized for 1000 steps using AUTOMINv1.0 without the inclusion of shell-waters [23].

### Extraction of networked salt bridges

**Figure 1:**
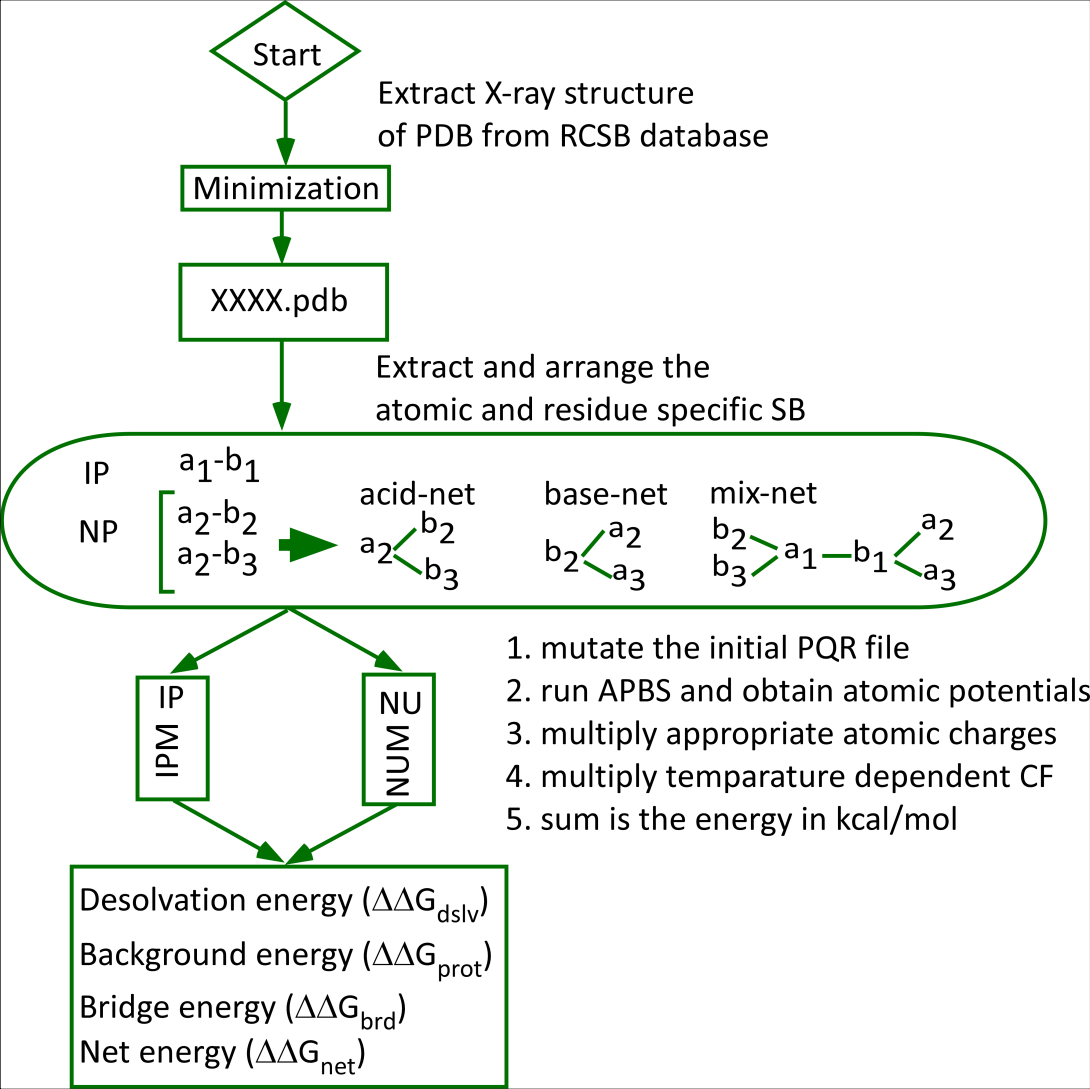
Flowchart for the computation of energy terms of saltbridges by isolated pair method (IPM) and network unit method (NUM). IP isolated pair; NU network unit; CF conversion factor. The positions of acidic and basic residues are indicated by the number.

Atomic and residue-specific isolated and networked salt-bridges are extracted from minimized crystal structure using SBION and SBION2 [27,28]. Three classes of networked salt-bridges are defined. First, acid networked salt-bridge is formed by acid with two or more base residue. Second, base networked salt-bridge is formed by base residues with two or many acid residues. Third, it is a mixed type salt bridge where acid and base networked saltbridges are interlinked together (Figure 1).

### Computation of energy terms of salt bridges

Poisson-Boltzmann Equation (PBE) solver i.e. APBS [20] was used along with PDB2PQR [21] for the determination of energy terms of IP (isolated pair) and NU (network unit) (Figure 1) using IPM [13,14] and NUM. PDB2PQR gives force-field dependent atomic charge (Q) and radius (R) file (PQR) of PDB. The initial PQR file was mutated using hydrophobic isosteres as per the requirement to obtain different energy terms (ΔGbrd, ΔGprot, and ΔGdslv). We followed IPM as earlier for isolated pairs of salt-bridge [13,14,24,25,26]. For networked salt-bridges (Figure 1), we used NUM, which differs from the direct application of IPM. In this computation, all partners that are present in a network unit (Figure 1) were taken into consideration for mutation of initial PQR file, run of the APBS and obtaining the energy in kcal/mol (Figure 1).

## Results

### Buried and networked salt-bridges in thermophilic protein

**Figure 2:**
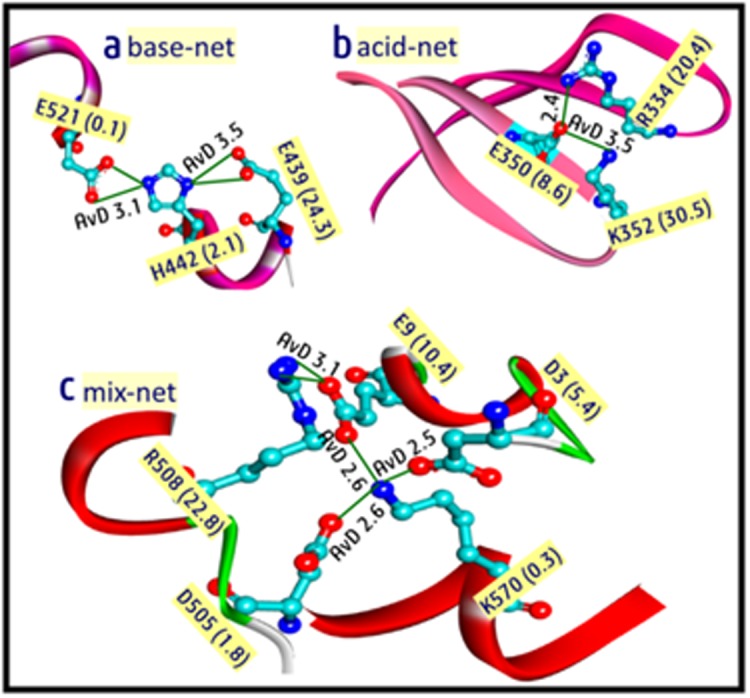
Buried and inter-helix base-network (a), inter-strand acidnetwork (b) and mix-network (c) salt-bridges. The residue number is shown with accessibility in Å^2^. AvD indicates average distance in Angstrom.

Typical salt-bridges that are investigated here are shown in Figure 2 along with average distance (AvD) and accessibility in the first bracket. Figure 2a is the base networked salt-bridge (base-net) where H442 is bonded with E439 and E521. Although E439 is exposed, the accessibility of H442 and E521 are much lower. This salt-bridge is the inter-helix type. Inter-strand acid-networked saltbridge (acid-net) is shown in Figure 2b, which associate AvD and accessibility parameters. The mixed type networked salt-bridge (mix-net, Figure 2c) is constituted by two basic and three acidic partners. Remarkably, all these partners are deeply buried except the R508, which is at the core-surface interface. This salt-bridge makes interconnection between three different helical regions.

### Desolvation cost is reduced by the formation of networked salt-bridge

Thermophilic proteins follow a number of strategies of which increase of salt-bridge forming residues (sbfrs) have been the prime factor [11]. A greater fraction of sbfrs form networked salt-bridges in the core and in the surface [11]. Do buried networked saltbridges contribute to the stability than an equivalent number of isolated salt-bridges? To check this, we have considered the following typical salt-bridges (Figure 2 and Table 1). Desolvation energy of acid (Ai) and base (Bj) are computed separately. For the folded state of the protein, only the CHARMM force field generated atomic partial charges of the side-chain of Ai/Bj were kept. Mainchain atoms (C, CA, N, H, HA, O) of Ai/Bj, and main and sidechains of other residues were mutated using hydrophobic isosteres. PQR (protein's charge and radius) file, thus generated, was subjected for manually configured multigrid Poisson-Boltzmann calculation under single Debay-Huckel boundary condition (mPBsDH) using APBS [20]. In the unfolded state of Ai/Bj, the main-chains of (i-1/j-1) and (i+1/j+1) residue were also associated with Ai/Bj as earlier [16]. APBS was run with mPBsDH. The atomic potential thus obtained, was multiplied by partial charges of the side-chain of Ai/Bj and then multiplied by 0.593 whose sum is the desolvation free energy of Ai/Bj in kcal/mol. The desolvation free energy of the network unit is the sum of that of the constituent acidic and basic partners (Table 1).

Table 1 shows base-networked, acid-networked and mixednetworked salt-bridges. The average ASA shows that each of this salt-bridge pair is present under buried conditions. In the mixednetworked type, the ASA values are seen to be very low. ΔΔGdslv for each of sbfrs was calculated using earlier formula and model [24,25]. The unit of desolvation cost is kcal/mol if not mentioned otherwise. The superscript in the residue is its position in protein's sequence.

The net-desolvation cost of IP or NU is generally computed in a pair-wise manner [11,14]. Here the desolvation cost for the network unit (NU) is obtained by summing the desolvation cost of each partner that constitutes the NU (Table 1). We see that as the partners in networked salt-bridge increases, the reduction of desolvation term is more. In base and acid networked salt-bridge, the desolvation term is reduced by one term as each of base-net and acid-net are composed of 3 partners with one common partner in them. Notably, in isolated pair form, common partner of salt-bridge gets multiple entries. In network unit, the common repeated energy term is removed and thus, the net desolvation cost of the networked unit is always lower than that in isolate pair form. It is seen that eight partners are forming a five-membered mix-net (Table 1). Here, desolvation terms are reduced from eight to five (Table 1). Overall, more the inter-linking in the network, the more is the reduction of desolvation cost.

### Computation of background energy for networked salt-bridge

ΔΔGprot for NU was computed using a similar method as IP [16,13]. Charges for the side-chains of all but partners of NU were mutated (Table 1). mPBsDH was solved. Atomic potential thus obtained is multiplied by 0.593 and atomic charges of side chains of [i] acidic (A), [ii] basic (B), [iii] polar (P) and [iv] non-polar (H) residues (except the ones that are present in the NU) to obtain background contributions due to acidic, basic, polar and non-polar parts respectively (Table 2).

By using different combinations (A+B or A+B+P or A+B+P+H), the contribution of different forms of the background energy term (charge or charge and polar or all) can be made [17,19,20]. To obtain the contribution of charged residues, we have to sum the ΔΔGprot(A) and the ΔΔGprot(B) terms (Table 3).

### Computation of bridge energy for networked salt-bridge

Different methods could be followed to obtain an accurate estimate of bridge energy of an NU. We followed the isolated pair method [16,13,14] for computation of ΔΔGbrd term of networked saltbridge. For example, for the base network (one base linked with multiple acids), partial atomic charges of the side-chain of base residue were retained and charges of all other residues were mutated by hydrophobic isosteres. Using this as the input file, mPBsDH was solved. The potential file is generated. Now the atomic partial charges of side-chains of acidic residues that are in the network unit, are multiplied with the corresponding potential and the constant i.e. 0.593. The sum represents the ΔΔGbrd for base networked salt-bridge in kcal/mol unit. For the acid network, a similar procedure was followed. However, in this case, instead of the base, atomic charges of side-chain of acidic residue were used to generate the potential file. Side-chains atomic charges of basic residue, atomic potential and the constant (0.593) were used to generate the bridge energy. For mix-net, we repeated the cycle over the number of base/acid residues in the mixed network. The events in cycle follow as i] generate potential using side-chain atomic charges of base/acid as input-file, ii] obtain the energy by multiplying the charges of side-chains of other residues present in the mixed network, the corresponding potential and the constant (0.593). Summing the energies of all cycles would give the accurate estimate of net bridge energy of the mixed-networked salt-bridge as has been verified using different methods [13,16].

## Discussion

### Salt-bridge forming residues are more in sequence and in the core of thermophilic proteins

Salt-bridge is specific electrostatic interaction between positive and negative charged residues that contribute to the overall stability of native protein [29,13,14]. It can either be isolated (IP) or network (NU) type. In isolated form, positive and negative charged partners participate in 1:1 ratio. On the other hand, networked salt-bridge involves more than one acidic or basic or both residues to form base-net (1 base: n acids), acid-net (1 acid: n bases) or mix-net (≥ 2 acids and ≥ bases; Table 2) type salt-bridge respectively. Where n is great than or equal to two. Analyses of binary items of a large database of crystal structures [27,28] showed that these saltbridges could either be in the core or in the surface [13]. In mesophilic proteins, the frequency of buried salt-bridge is very less. In a 150 residue protein, only one pair has been seen to be under buried condition [30]. On the other hand, buried salt-bridges are shown to be more frequent in thermophilic homologues [11]. Analysis of the core and surface composition on a number of proteins showed that core harbors 10-20% polar and charged residues [31,32]. In mesophilic proteins, while the hydrophobic force is the determinant of the fast-step of protein folding [29], the formation of buried salt-bridge is in the rate-limiting step [18]. Our earlier work on the thermophilic protein (5T88) showed that although the level of hydrophobic residues is similar as mesophiles, charged residues increases in the sequence and in the core of the former relative to the latter. Further, 75% of the homologous positions of the protein undergo substitutions that favor formation of salt-bridges. Taken together, it appears weak interactions that contribute to the overall stability of mesophilic proteins are somewhat modulated in the case of thermophilic homologues.

### Energy terms of the networked unit are not directly obtained by the isolated-pair method

The contribution of buried and networked salt bridges in the stability of protein has been much controversy in recent time, which could be understood from the dielectric constant of the medium [29]. Thermophilic proteins harbor high frequency of buried and networked salt-bridges [33] and thus, at mesophilic conditions (at low temperature and high dielectric medium) these proteins become non-functional [6]. In turn, in thermophilic conditions (at high temperature and low dielectric medium), hydrophobic force is weak. Under this condition, the core structure of thermophilic proteins may require additional stabilizing force. While lone charges in the core of protein would be highly destabilizing, salt-bridge could stabilize it [29]. However, saltbridge to be stable required to be located in the surface of the protein [29]. At this point, it is worth raising the question that is buried salt-bridge stable. The work of Hendsch and Tredor (1994) showed that ΔΔGnet of buried salt-bridge is largely costly due to high desolvation cost [19]. Further, the networked salt bridge was demonstrated to be more prone to be unstable than its constituent pairs [16]. Instead, if such design is replaced by hydrophobic isosteres, the overall stability of protein increases [16,17]. Oppositely, using Poisson-Boltzmann Equation (PBE) along with the earlier method [16], it has been demonstrated that a large number of buried salt-bridges (both isolated and networked) are highly stable in thermophilic as well as mesophilic glutamate dehydrogenases [11]. Although, methodologically similar, in the earlier case the force field was CHARMM [16] and in the latter, it was PARSE3 [11].

In all the above methods, energy terms of the salt bridge are obtained as isolated pair method (IPM) and the energy terms for networked salt-bridges are then obtained simply by summing the energy terms of isolated pairs that are belonging to a network unit (NU). The following concern may arise in this context. First, the actual energetic contribution of the desolvation cost for a network salt-bridge would be overestimated. Second, background energy term for a networked salt-bridge will also be overestimated or underestimated based on the composition of the microenvironment of the network unit. Third, the desolvation cost and the background energy terms could be erroneous due to the inclusion of additional residue in background, which is otherwise present in a networked unit. The fact that in thermophilic protein, different forms of networked salt-bridges are more frequent under buried conditions [33,34], and buried salt-bridge are long been demonstrated to be unstable due to very high desolvation cost, the question appears as to is there an evolutionary benefit for the formation of these saltbridges under buried condition. Following the present method (NUM), we show that more the intricacy of a networked salt bridge, the lesser would be the desolvation cost. For example, in acid-net and base-net (Table 1 and Table 2), as in each case, there is one common residue in the networked unit, the desolvation cost is reduced for the common residue. In the case of mix-net, K570 and E9 are common in the formation of a networked unit from 4 isolated pairs. Thus, the desolvation cost due to two of K570 and one E9 are to be subtracted from total desolvation cost (Table 1 and Table 2). A similar correction is also necessarily required for the computation of background energy terms (Table 1 and Table 3). Taken together, it is apparent that the computation of net and component energy terms for the network unit is not directly obtained from that of the isolated pair method (Table 3).

### The implication of buried and networked salt-bridge in thermophilic protein

Buried salt-bridges have been largely unstable due to high desolvation cost [29,16,18,19]. Presence of isolated charge is more destabilizing than an ion-pair [16]. The existence of the former in the core is more as the latter is denser than that of the surface. Thus, the formation of networked salt-bridge is a way to circumvent such additional instability in the core of the protein. Is there any energetic advantage of the formation of networked salt-bridge in the core? A higher proportion of buried and networked salt-bridges are present in hyperthermophilic proteins [33,34]. Although it has been demonstrated, such salt bridges are more stable than its mesophilic homologue [11], the net and component energy terms are not computed using these networked salt-bridges as a unit. Application of NUM reveals that the high frequent buried and networked salt bridges in hyperthermophilic protein is justified as the reduction of desolvation cost is related with the intricacy of the NU. At high temperature and also at other extreme of physical and chemical conditions, as solvent properties drastically reduced the dielectric properties [8, 9], and as hydrophobic interactions are affected severely at low dielectric medium, evolutionary installation of buried and networked salt-bridges seems to have great implications for the maintenance of structure and stability of proteins from these microbes.

## Conclusion

The desolvation cost of BNSB is difficult to be compensated by other favorable energy terms. Yet such salt-bridges have been found to be more frequent in thermophilic proteins. It has been shown that these salt-bridges make stabilizing contributions in thermophilic proteins using isolated-pair method along with PARSE3 force field. Results show that the desolvation cost decrease as the candidates in a network unit increase. It should also be noted that other microenvironment features of the partners in the networked unit also have a role to play in thermostability.

## Figures and Tables

**Table 1:** : Computation of desolvation cost of acid, base, and mixed type networked salt bridges. Salt-bridge (SB) pairs, average accessibility (Av. ASA), network unit and net desolvation cost (Net ??Gdslv) are shown.

SB pairs	Av ASA Å2	Network unit	ΔΔGdeslv
			kcal/mol
H442-E439	13.6	E439-H442-E521	17.4
H442-E521	1.3	(base-net)	
R334-E350	14.5	R334-E350-K352	10
K352-E350	19.6	(acid-net)	
K570-D3	3	(D3)(D505)-K570-E9-R508	34.4
K570-D505	0.6	(mix-net)	
K570-E9	5.3		
R509-E916.1		

**Table 2:** Partitioning of background energy terms into acidic (A), basic (B), polar (P) and non-polar (H) parts along with the total. The details of the computation of background energy for a given networked salt-bridge are shown in the text.

	Partition of background energy Kcal/mol				Total Kcal/mol
Networked unit	ΔΔGprot(A)	ΔΔGprot(B)	ΔΔGprot(P)	ΔΔGprot(H)	ΔΔGprot(Total)
					
Base net: E439-H442-E521	5.89	-15.2	-3.6	-0.99	-13.9
Acid net: R334-E350-K352	-12.2	2.18	0.32	0.22	-9.48
Mixed net: (D3)(D505)-K570-E9-R508	3.64	-15.1	-6.02	-2.2	-19.4

**Table 3:** Component and net energy terms of base-net, acid-net and mixed net along with details on the average accessibility, bond-multiplicity, and average bond distance.

SB pairs	ΔΔGbrd	ΔΔGprot *	ΔΔGdeslv	ΔΔGnet	Av. ASA (Å^2^)	mu	Av. Dist (Å)
Base-net	-20.6	-9.31	17.4	-12.51	8.8	4	3.275
Acid-net	-13.06	-10.02	10	-13.08	19.8	2	3.005
Mixed-net	-58.41	-11.46	34.4	-35.47	8.14	5	2.735
*only acid and base terms are considered; ASA accessibility; mu bond-multiplicity; Av. Dist average distance;
